# Cellular stress due to impairment of collagen prolyl hydroxylation complex is rescued by the chaperone 4-phenylbutyrate

**DOI:** 10.1242/dmm.038521

**Published:** 2019-06-20

**Authors:** Roberta Besio, Nadia Garibaldi, Laura Leoni, Lina Cipolla, Simone Sabbioneda, Marco Biggiogera, Monica Mottes, Mona Aglan, Ghada A. Otaify, Samia A. Temtamy, Antonio Rossi, Antonella Forlino

**Affiliations:** 1 Department of Molecular Medicine, Biochemistry Unit, University of Pavia, 27100 Pavia, Italy; 2 Istituto Universitario di Studi Superiori - IUSS, 27100 Pavia, Italy; 3 Istituto di Genetica Molecolare, Consiglio Nazionale delle Ricerche, 27100 Pavia, Italy; 4 Department of Biology and Biotechnology, University of Pavia, 27100 Pavia, Italy; 5 Department of Neuroscience, Biomedicine and Movement, University of Verona, 37134 Verona, Italy; 6 Department of Clinical Genetics, Human Genetics & Genome Research Division, Center of Excellence for Human Genetics, National Research Centre, Cairo 12622, Egypt

**Keywords:** Osteogenesis imperfecta, Endoplasmic reticulum stress, Chemical chaperone, Unfolded protein response, 4-PBA

## Abstract

Osteogenesis imperfecta (OI) types VII, VIII and IX, caused by recessive mutations in cartilage-associated protein (*CRTAP*), prolyl-3-hydroxylase 1 (*P3H1*) and cyclophilin B (*PPIB*), respectively, are characterized by the synthesis of overmodified collagen. The genes encode for the components of the endoplasmic reticulum (ER) complex responsible for the 3-hydroxylation of specific proline residues in type I collagen. Our study dissects the effects of mutations in the proteins of the complex on cellular homeostasis, using primary fibroblasts from seven recessive OI patients. In all cell lines, the intracellular retention of overmodified type I collagen molecules causes ER enlargement associated with the presence of protein aggregates, activation of the PERK branch of the unfolded protein response and apoptotic death. The administration of 4-phenylbutyrate (4-PBA) alleviates cellular stress by restoring ER cisternae size, and normalizing the phosphorylated PERK (p-PERK):PERK ratio and the expression of apoptotic marker. The drug also has a stimulatory effect on autophagy. We proved that the rescue of cellular homeostasis following 4-PBA treatment is associated with its chaperone activity, since it increases protein secretion, restoring ER proteostasis and reducing PERK activation and cell survival also in the presence of pharmacological inhibition of autophagy. Our results provide a novel insight into the mechanism of 4-PBA action and demonstrate that intracellular stress in recessive OI can be alleviated by 4-PBA therapy, similarly to what we recently reported for dominant OI, thus allowing a common target for OI forms characterized by overmodified collagen.

This article has an associated First Person interview with the first author of the paper.

## Abstract (precis)

**Editor's choice:** Mutations in the collagen 3-prolyl hydroxylation complex cause a cellular stress that is rescued by the chaperone ability of 4-phenylbutyrate.

## INTRODUCTION

Osteogenesis imperfecta (OI) is a collagen-related heritable disorder affecting several connective tissues, but is mainly characterized by skeletal deformity and bone fragility (Marini et al., 2017). Together with the dominant forms caused by mutations in type I collagen and representing over 85% of OI cases, recessive and X-linked OI have been described since 2006. These forms are characterized by defects in proteins involved in collagen type I folding, post-translational modifications, intracellular trafficking, extracellular processing or osteoblasts maturation (Forlino and Marini, 2016; Lindert et al., 2016).

Synthesis of type I collagen includes a complex intracellular and extracellular series of events preceding mature collagen fibril formation and involves several molecular players. Briefly, two proα1 and one proα2 chains are synthesized in the endoplasmic reticulum (ER) and linked in a trimeric molecule thanks to specific C-terminal recognition sequences and covalent disulfide bridges occurring in close proximity to the ER membrane. During their translation and before triple-helical folding, the α-chains undergo various post-translational modification events, including hydroxylation of proline in C-4 and C-3 and of lysine residues (Ishikawa and Bächinger, 2013). Proline-4 hydroxylation, catalyzed by prolyl-4-hydroxylase B (P4HB), affects almost all the proline residues placed in the Y position of the collagen triplet unit (Gly-X-Y). 4(R)-hydroxy-L-proline (4-Hyp) residues are fundamental for helix stability by favoring water-bridged intramolecular hydrogen bonding. The hydroxylation of triple helical and telopeptide lysine residues, performed by lysyl hydroxylase 1 and lysyl hydroxylase 2, respectively, provides the substrates for successive intracellular glycosylation and extracellular covalent crosslink formation. The role of 3(S)-hydroxy-L-proline (3-Hyp) instead is still poorly defined (Hudson and Eyre, 2013; Pokidysheva et al., 2014). Very few proline residues in collagen type I are 3-hydroxylated, likely excluding their role in collagen stability (Marini et al., 2007). In α1(I), only Pro986 is always present as 3-Hyp and this post-translational modification is performed by prolyl-3-hydroxylase 1 (P3H1) that is associated in a 1:1:1 ratio with cartilage-associated protein (CRTAP) and cyclophilin B (CyPB) to form a complex active in the ER (Ishikawa et al., 2009). The relevance of 3-Hyp in collagen folding as well as in proper fibril formation was proposed following the identification of three recessive OI forms characterized by the lack of α1(I)Pro986 C-3 hydroxylation and caused by mutations in one of the three genes encoding the proteins of the ER complex (Marini et al., 2007). Defects in CRTAP, the helper protein of the complex, are responsible for OI type VII (OMIM # 610682), and patients show a moderate to lethal phenotype with growth deficiency, rhizomelia, severe osteoporosis and neonatal fractures (Morello et al., 2006). OI type VIII (OMIM # 610915) is the consequence of mutations in P3H1, the protein of the complex that catalyzes α1(I)Pro986 C-3 hydroxylation. OI type VIII patients usually show a severe to lethal phenotype with the symptoms overlapping those of type VII (Cabral et al., 2007). Importantly, CRTAP and P3H1 are mutually stabilizing in the ER (Chang et al., 2010).

Mutations in *PPIB* are responsible for OI type IX (OMIM # 259440). *PPIB* encodes for CyPB, the peptidyl-prolyl *cis-trans* isomerase that catalyzes the isomerization of the peptide bonds involving proline residues, the rate-limiting step reaction in collagen folding. The phenotype of OI type IX patients ranges from moderate to lethal, partially overlapping OI type VII and VIII forms, but without rhizomelia (Barnes et al., 2010; van Dijk et al., 2009).

The absence of CRTAP, P3H1 and CyPB, associated with complete lack or reduced α1(I)3-Hyp986, delays collagen type I folding, causing overmodification of the helical region and decreased collagen secretion at least in OI dermal fibroblasts (Marini et al., 2007). A still open question to understand the molecular basis of these OI recessive forms is whether the OI phenotype is caused by the absence of 3-Hyp in the bone matrix or by a defect in intracellular collagen folding and secretion, or a combination of both. Interestingly, in a knock-in mouse in which the P3H1 catalytic site was inactivated, but the enzyme was still able to complex with CRTAP, a mild bone phenotype was present (Homan et al., 2014).

The overmodified collagen molecules secreted in the extracellular matrix (ECM) in OI type VII, VIII and IX assemble in irregular fibrils, which impair proper mineralization, affecting bone properties, but their intracellular effects are still unknown (Forlino et al., 2011). Interestingly, using a functional proteomic approach on lysates obtained from primary fibroblasts of patients with mutations in *CRTAP*, *P3H1* or *PPIB*, we demonstrated an altered cytoskeleton and altered nucleoskeletal assembly, pointing to an impairment of the intracellular compartment (Gagliardi et al., 2017).

No effective therapy is available for any of the OI forms, and bisphosphonates, the most commonly used drugs, are anti-catabolic molecules that impair osteoclast activity and bone remodeling, improving bone mineral density, but without positive effects on bone properties (Besio and Forlino, 2015). Thus, the search for new and, likely, common pharmacological targets for multiple OI forms is an urgent patient need. We used seven primary fibroblast lines obtained from recessive OI type VII, VIII and IX patients to evaluate how cells react to the presence of overmodified collagen because of mutations in the components of the collagen prolyl-3-hydroxylase complex. We demonstrated that mutant collagen accumulates in the ER, causing unfolded protein response (UPR) activation and apoptotic death. We proved that the administration of the chemical chaperone 4-phenylbutyrate (4-PBA) ameliorates cellular homeostasis by mainly favoring protein secretion.

## RESULTS

Primary fibroblasts from seven previously described (*PPIB*) recessive OI patients with mutations in the components of the 3-hydroxylation complex were selected for the study. Three patients carry mutations in *CRTAP* (CRTAP-1, CRTAP-2 and CRTAP-3), three in *P3H1* (P3H1-1, P3H1-2 and P3H1-3) and one in *PPIB* (CyPB) (Table 1)*.*

**Table 1.**
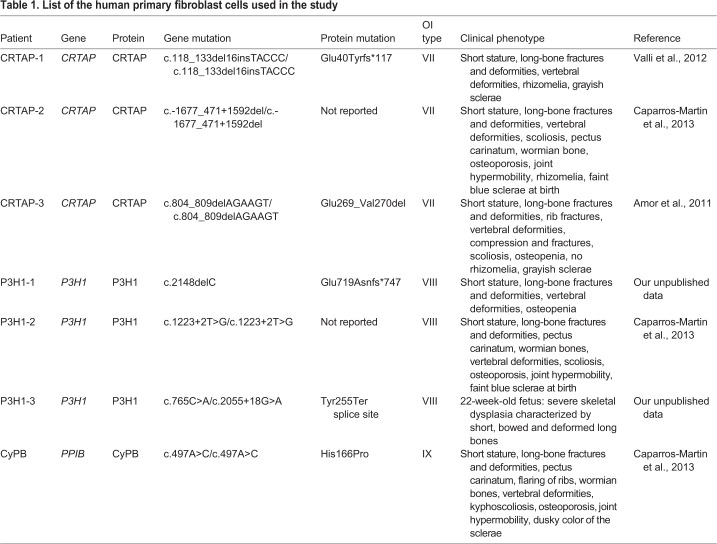
**List of the human primary fibroblast cells used in the study**

**Fig. 1.**
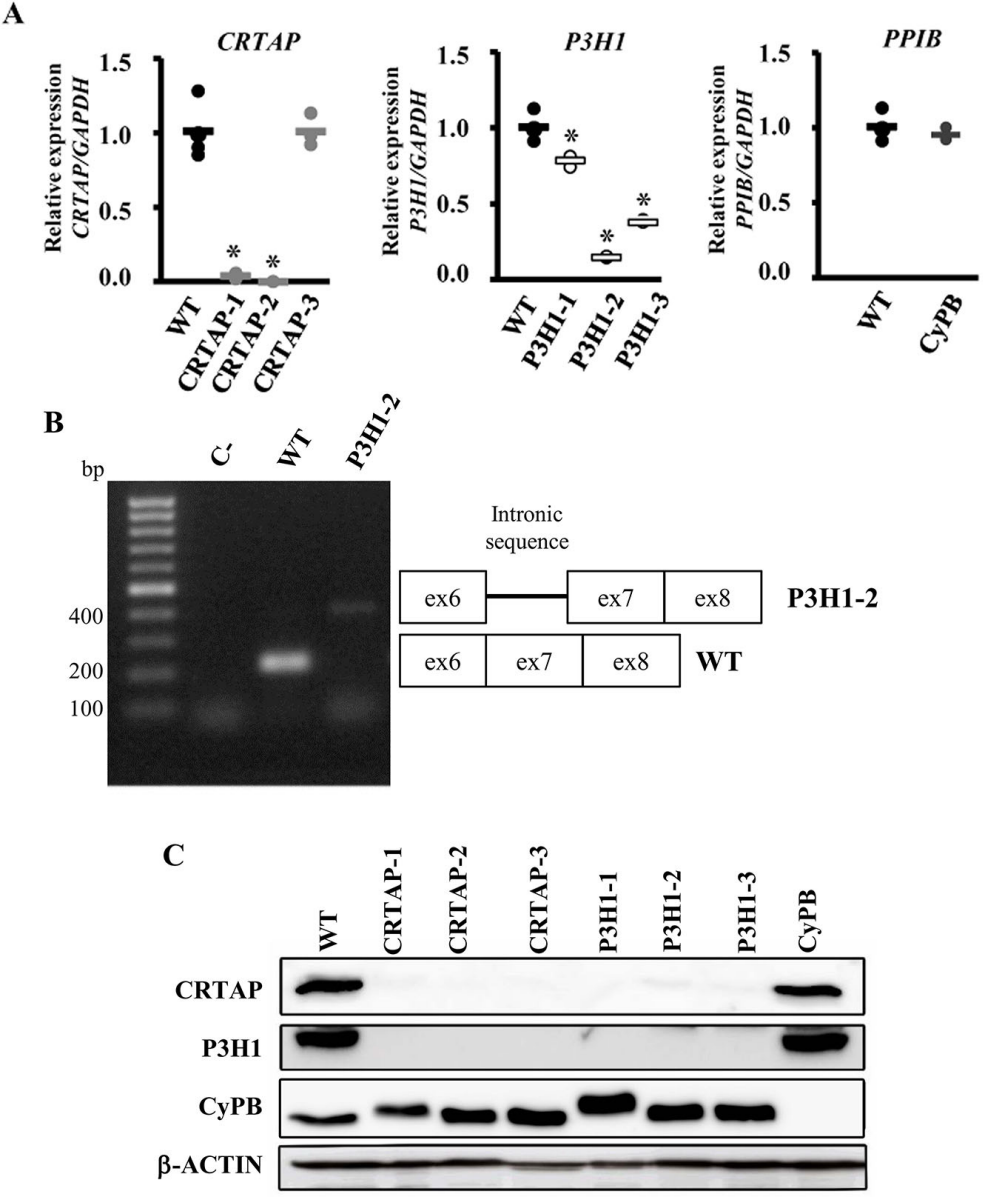
**Loss of mutant CRTAP, P3H1 and CyPB in OI patient fibroblasts.** (A) Quantitation of *CRTAP*, *P3H1* and *PPIB* expression evaluated by qPCR. Mutations in *CRTAP*, *P3H1* and *PPIB* caused a close to complete absence of the mutated transcripts in CRTAP-1, CRTAP-2 and P3H1-2 patients, and a reduced mRNA level in P3H1-1 and P3H1-3. *P<0.05. WT values are represented as black dots; CRTAP as gray dots; P3H1 as white dots; CyPB as dark gray dots. (B) Amplification of the exon 6-exon 8 region of *P3H1* transcript generated the expected 217 bp amplicon in control cells (WT), whereas, in the P3H1-2 patient, the presence of a higher molecular weight (∼400 bp) band compatible with intronic retention was detected. C-, RT-PCR negative control. (C) Representative western blot to evaluate the expression of CRTAP, P3H1 and CyPB in control (WT) and mutant cell lysate fractions (CRTAP-1, CRTAP-2, CRTAP-3, P3H1-1, P3H1-2, P3H1-3, CyPB). Loss of the mutated protein in patient's cells was demonstrated. Patients with mutations in *CRTAP* showed also no P3H1 expression and patients with mutations in *P3H1* showed no CRTAP expression, as a consequence of their mutual protection in the complex.

In CRTAP-1, in which the mutation is predicted to cause a frameshift resulting in a premature stop codon, and in CRTAP-2, in which a large genomic deletion including exon 1 was described, a strongly reduced (0.036±0.019) and no CRTAP expression, respectively, were detected by quantitative real-time PCR (qPCR), suggesting the activation of nonsense-mediated decay (Fig. 1A). Similarly, a reduced P3H1 expression (0.146±0.03) was present in P3H1-2 cells carrying an intronic mutation in intron 7, predicted to impair normal splicing. Indeed, no exon 6-8 amplicon was detected by reverse-transcription PCR (RT-PCR), but a band with higher molecular weight, compatible with the retention of the intronic sequence, was detected (Fig. 1B).

A reduction of about 50% of *P3H1* transcript was demonstrated in P3H1-3, a compound heterozygous for an allele carrying a missense mutation and a second allele predicted to impair the translation of the KDEL ER-retention signal. The defect in the P3H1-1 patient, the only one not molecularly characterized yet, was identified as a single-nucleotide deletion (c.2148delC) in *P3H1* exon 15. The mutation causes a frameshift and the introduction of a premature stop codon at position 747 (Glu719Asnfs*747). Only a slightly reduced *P3H1* expression (0.78±0.03) was detected (Fig. 1A). As expected, no impairment of CRTAP expression was found in CRTAP-3, carrying the homozygous deletion of 6 nucleotides (nt) responsible for the in frame removal of amino acids Glu269 and Val270, or in CyPB, carrying a homozygous single base-pair substitution generating the His166Pro in CyPB (Fig. 1A).

At the protein level, all cells from patients carrying mutations in *CRTAP* showed the absence of both CRTAP and P3H1 expression and, similarly, patients with mutations in *P3H1* showed no P3H1 and CRTAP expression, as expected given the mutual protection of these proteins in the complex (Chang et al., 2010). By contrast, the level of the third component, CyPB, was not affected (Fig. 1C). No CyPB expression was detectable in *PPIB* mutant cells despite normal transcript level, but the level of CRTAP and P3H1 proteins were within the normal range (CRTAP 1.00±0.19; P3H1 1.00±0.28).

### Mutations in the components of the prolyl 3-hydroxylation complex impair collagen structure and cell survival

**Fig. 2.**
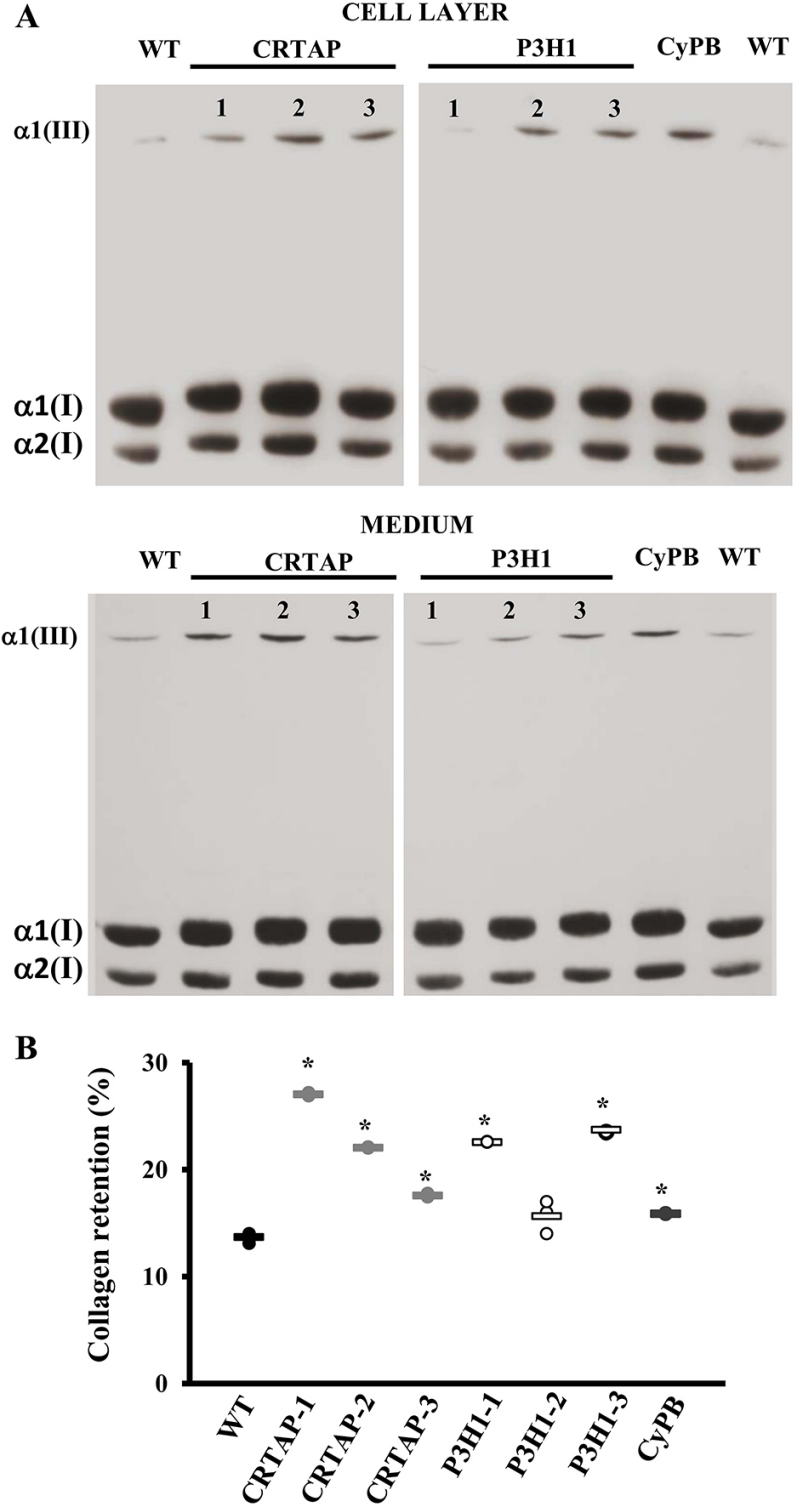
**Mutations in the collagen prolyl-3-hydroxylation complex lead to collagen overmodifications and collagen intracellular retention.** (A) Representative SDS-urea-PAGE fluorographies of ^3^H-labeled collagen extracted from the cell layer and medium of control (WT) and patient (CRTAP-1, CRTAP-2, CRTAP-3, P3H1-1, P3H1-2, P3H1-3, CyPB) fibroblasts. In mutant samples, broader and slower α(I) bands demonstrated the overglycosylation of type I collagen. (B) The percentage of intracellular collagen retention was evaluated as a ratio between the CPM in the cell layer and in medium plus cell layer. Collagen molecules in mutant cells were more intracellularly retained compared to WT. *P<0.05. WT values are represented as black dots; CRTAP as gray dots; P3H1 as white dots; CyPB as dark gray dots.

The impairment of the 3-hydroxylation complex is known to affect type I collagen folding, causing its increased hydroxylation and glycosylation (Forlino and Marini, 2016). In all analyzed OI cells, the presence of collagen overmodification was confirmed by electrophoretic analysis of ^3^H-labeled type I collagen. Steady-state collagen gels revealed the typical broadening of the α(I) bands in both cell-layer and medium fractions (Fig. 2A). Furthermore, an increase of collagen retention was detected in mutant cells compared to controls, and kinetic analysis showed a decrease in collagen secretion (Fig. 2B and Fig. S1).

Electron microscopy imaging revealed the presence of large vacuoles, resembling autophagosome vesicles since double membranes were occasionally detectable, and the ER cisternae were clearly enlarged compared to control cells. The ER looked normal in P3H1-2 cells (Fig. 5B).

**Fig. 3.**
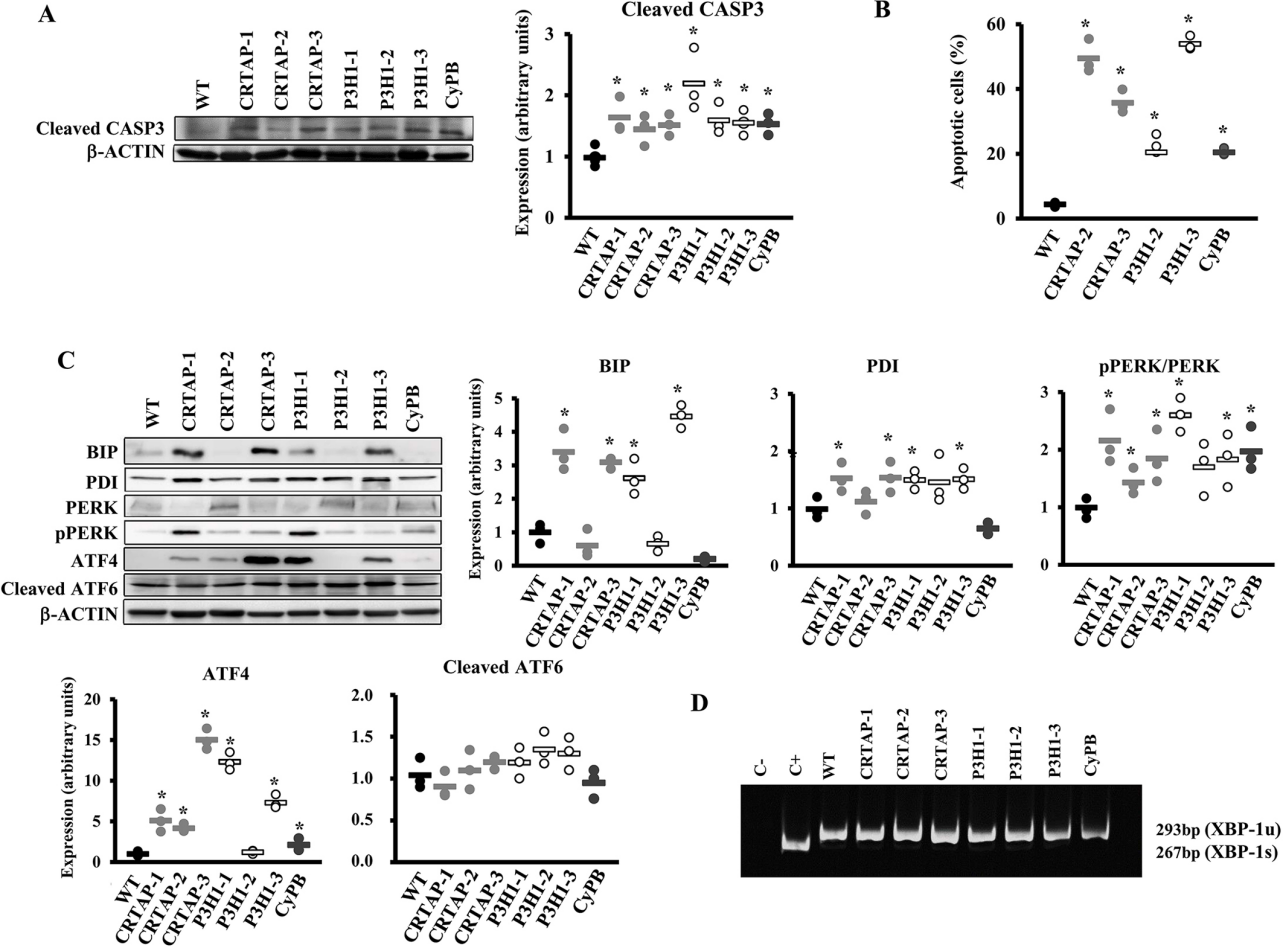
**Activation of apoptosis and the UPR in recessive OI patient fibroblasts.** (A) Representative western blot (left) to evaluate the expression of cleaved caspase 3 (CASP3), a terminal marker for apoptosis and the dot plot of the quantitation analysis (right). β-actin was used for normalization. (B) Quantitative analysis of the fraction of apoptotic events in the cell lines following FACS analysis upon cells staining with annexin V (FITC) and propidium iodide (PI). Apoptosis is activated in all tested OI patients' cells. (C) Representative western blots (left) and dot plots of the quantitative analysis (right and bottom) of the collagen chaperone PDI and of proteins involved in the UPR (BIP, PERK, p-PERK, ATF4, ATF6) in control (WT) cells and in cells with mutations in CRTAP, P3H1 or CyPB. The PERK branch of the UPR was upregulated in all patients' fibroblasts with the exception of patient P3H1-2. β-actin was used for normalization. WT values are represented as black dots; CRTAP as gray dots; P3H1 as white dots; CyPB as dark gray dots. *P<0.05. (D) RT-PCR amplification of *XBP1* mRNA from control (WT) and patient cells. The spliced XBP1-1s form of XBP1 transcript (XBP-1u) is not detectable in patient cells. Fibroblasts treated with thapsigargin were used as positive control (C+).

Apoptosis occurrence was demonstrated in all OI mutant cells by the increased level of cleaved caspase 3 (Fig. 3A) and confirmed by fluorescence activated cell sorting (FACS) upon annexin V/Dead-positive cell labeling. Indeed, an higher percentage of apoptotic cells compared to controls (4.31±0.78%) was detected by FACS in CRTAP-2, CRTAP-3, P3H1-2, P3H1-3 and CyPB fibroblasts (49.00±5.2%, 35.74±3.57%, 22.86±2.83%, 53.93±2.17 and 20.42±1.11%, respectively) (Fig. 3B and Fig. S2).

### UPR is activated in fibroblasts from patients with recessive OI

Given the intracellular presence of overmodified collagen molecules in the recessive patients' fibroblasts, we investigated the expression of the chaperones binding immunoglobulin protein (BIP) and protein disulfide isomerase (PDI) and the activation of the three branches of the UPR: the eukaryotic translation initiation factor 2 alpha kinase 3 (PERK) branch, the inositol-requiring enzyme 1α (IRE1 α) branch and the activating transcription factor 6 (ATF6) branch.

Four out of seven cell lines showed an increased level of both BIP, the best-characterized activator of the UPR sensors, and PDI, which catalyzes the formation and isomerization of disulfide bonds necessary for protein native state and which is known to interact with single collagen α chains (Fig. 3C) (Wilson et al., 1998).

The phosphorylated PERK (p-PERK):PERK ratio was significantly increased in all mutant cells with the exception of P3H1-2, in which a trend was detectable. Consistently, in these cell lines the expression of activating transcription factor 4 (ATF4), the effector of p-PERK, was also increased (Fig. 3C), confirming the activation of the UPR branch. No activation of the ATF6 and IRE1α branches was identified since no difference in cleaved ATF6 was detected and the IRE1α-mediated splicing of *XBP1* in mutants was comparable to controls (Fig. 3D).

Based on these data, we demonstrated that mutations in the prolyl-3-hydroxylation complex, causing the synthesis of overmodified collagen, lead to the disruption of ER homeostasis and consequent activation of the PERK branch of the UPR in OI fibroblasts.

### Recessive OI fibroblasts react to cellular stress by activating autophagy

**Fig. 4.**
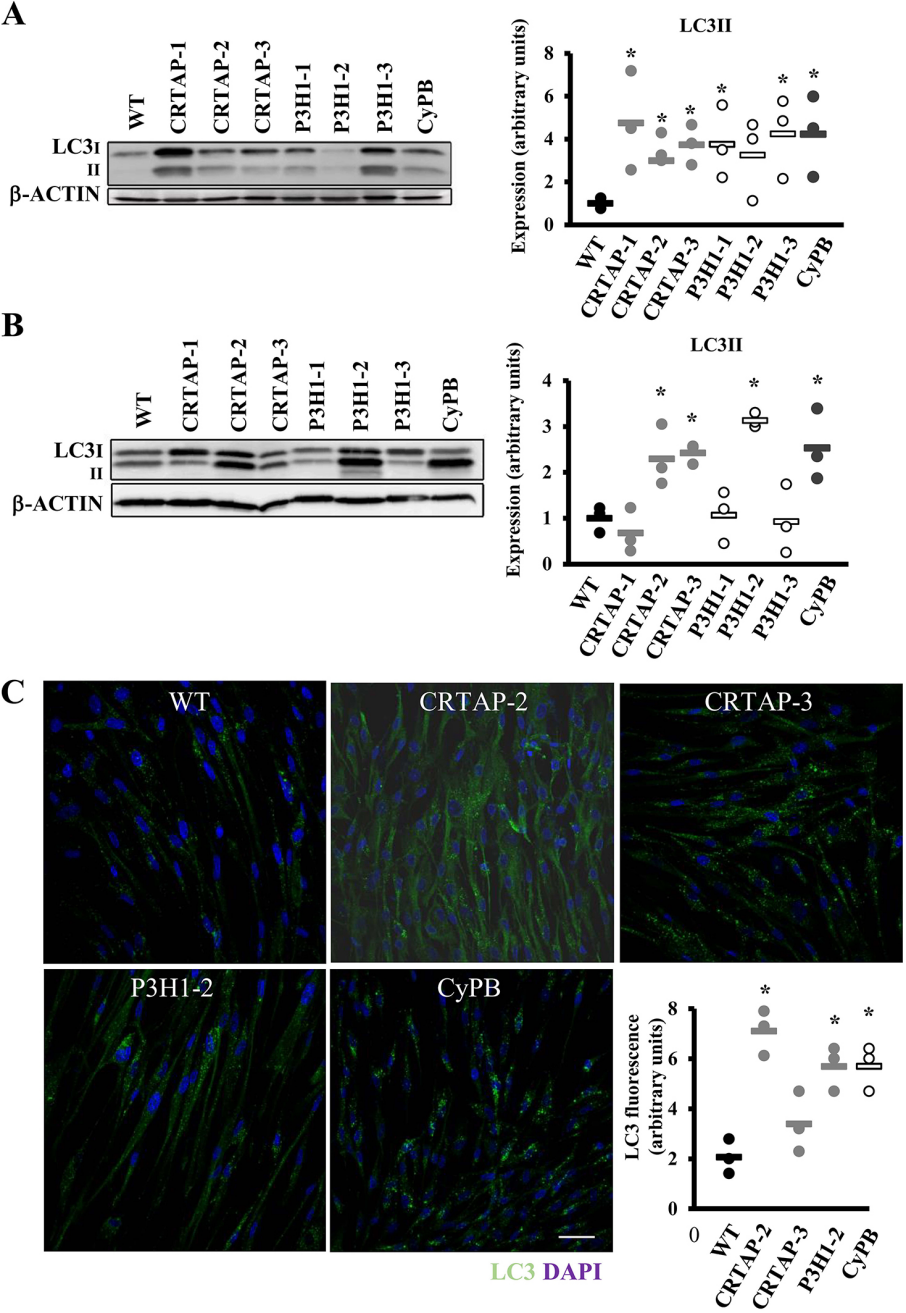
**Recessive OI cells react to cellular stress by activating autophagy.** (A) Representative western blot (left) and dot plot of the quantitative analysis (right) of the terminal autophagic marker LC3 in control (WT) and in cells with mutations in CRTAP, P3H1 or CyPB. LC3-II is upregulated in all cases except in patient P3H1-2. β-actin was used for normalization. (B) Representative LC3 western blot (left) performed on cell lysates obtained following chloroquine incubation from WT and mutant samples, and dot plot of the quantitative analysis (right). The terminal marker of autophagy evaluated in dynamic conditions is increased in CRTAP-2, CRTAP-3, P3H1-2 and CyPB. β-actin was used for normalization. (C) Representative LC3 immunofluorescence images of WT and mutant fibroblasts treated with chloroquine. Quantitation of the total area of punctate signal per cell confirms the activation of autophagy. DAPI (nuclei) in blue and LC3 in green. Magnification 40×, zoom 4×. WT values are represented as black dots; CRTAP as gray dots; P3H1 as white dots; CyPB as dark gray dots. *P<0.05. Scale bar: 40 µm.

Given the presence and/or intracellular accumulation of overmodified collagen molecules and with autophagy being the first cell response to constitutive dysfunctional cellular components, its activation was investigated, evaluating the expression of the terminal autophagic marker, the microtubule-associated protein 1A/1B-light chain 3 (LC3-II). The expression of LC3-II was upregulated in all cases except in patient P3H1-2 (Fig. 4A). Following chloroquine treatment, the expression of LC3-II was increased compared to wild type (WT) in CRTAP-2, CRTAP-3, P3H1-2 and CyPB cells, indicating a general accumulation of LC3-II due to the block in autophagic flux (Fig. 4B). The quantitation of LC3 immunofluorescence in OI fibroblasts treated with chloroquine was performed to validate the activation of the autophagic pathway by an independent assay. As expected, the LC3 signal was significantly increased compared to controls in CRTAP-2, CRTAP-3, P3H1-2 and CyPB cells, in agreement with the western blot data (Fig. 4C).

### 4-PBA ameliorates recessive OI fibroblasts homeostasis

**Fig. 5.**
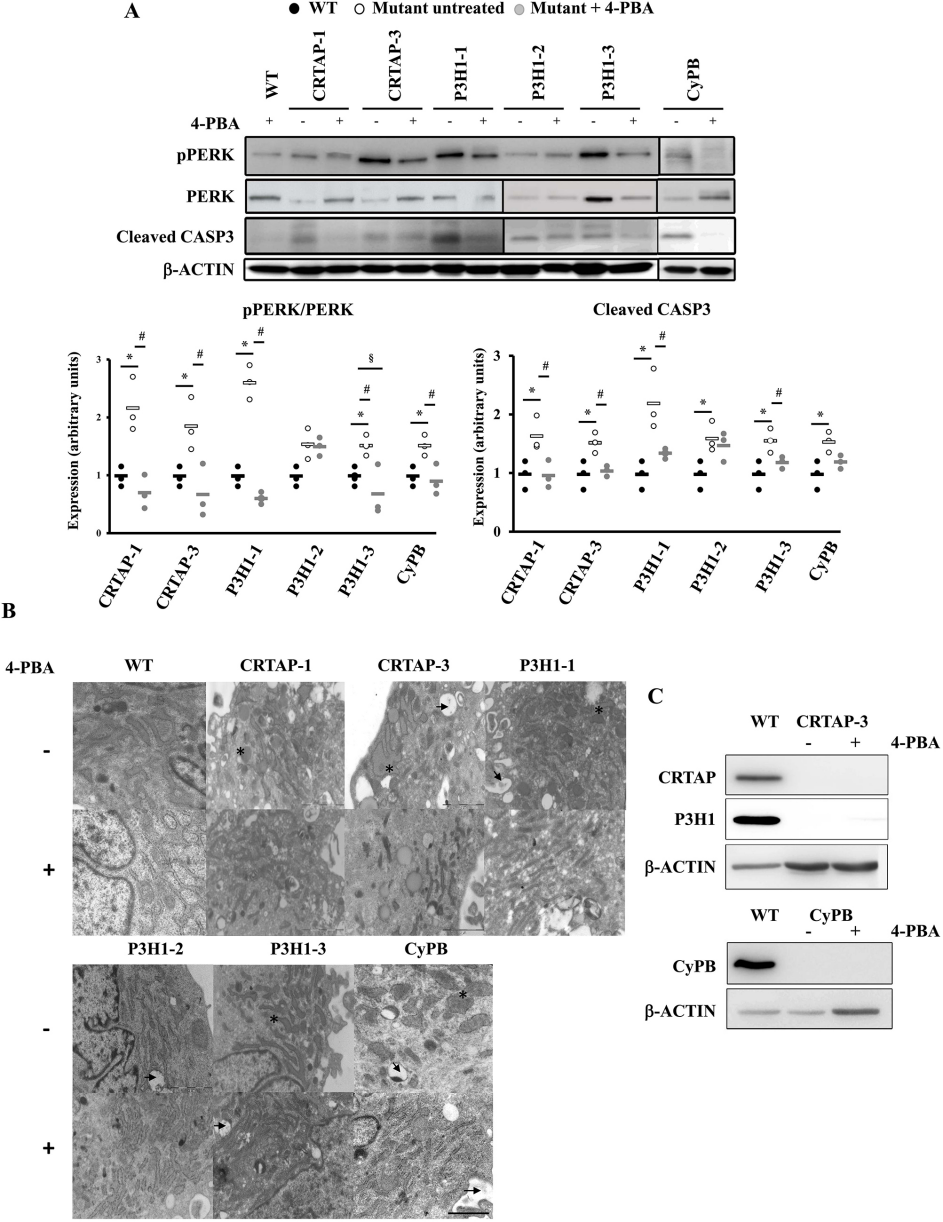
**4-PBA ameliorates recessive OI fibroblast homeostasis.** (A) Representative western blots (top) and dot plot of the quantitative analysis (bottom) of p-PERK and cleaved caspase 3 (CASP3) in the absence (−) or presence (+) of 4-PBA incubation in control (WT) cells and in cells with mutations in CRTAP, P3H1 or CyPB. The levels of these proteins were compared in treated versus untreated cells and in treated OI cells versus untreated controls. β-actin was used for normalization. *P<0.05 mutant fibroblasts with respect to control fibroblasts. ^#^*P*<0.05 treated mutant fibroblasts with respect to untreated mutant fibroblasts. ^§^*P*<0.05 treated mutant fibroblasts with respect to untreated control fibroblasts. p-PERK:PERK and cleaved caspase 3 levels were decreased to or even less than control values in all cases, with the exception of P3H1-2. WT untreated values are shown as black dots, mutants untreated as white dots and mutants treated as gray dots. (B) Transmission electron microscopy representative images of OI patient fibroblasts in the absence (−) or presence (+) of 4-PBA. The analyses revealed ER enlargement (*) and cellular vacuolization (arrow) in mutant cells with the exception of P3H1-2. 4-PBA treatment reduced the ER cisternae enlargement. Magnification 20,000×. (C) Representative western blot of CRTAP and P3H1 in control (WT) and CRTAP-3 cell lysates, and of CyPB in WT and CyPB lysates. No protein rescue was detected after the treatment in the two mutant cell lines, in which normal transcript level was detected. β-actin was used for normalization. Scale bar: 2 µm.

To alleviate cellular stress due to intracellular retention of overmodified collagen molecules, patient fibroblasts and control cells were treated with 4-PBA, a well-known chemical chaperone, FDA-approved as an ammonia scavenger for urea cycle disorders (Matoori and Leroux, 2015). The effect of the drug was evaluated following the activation of the PERK branch of the UPR and the activation of caspase 3, as a signature for apoptosis, by western blotting. Their levels were compared in control and OI treated versus untreated cells and in treated OI cells versus untreated controls. None of the selected markers was significantly changed in WT after 4-PBA administration (data not shown). Interestingly, following the treatment, p-PERK:PERK and cleaved caspase 3 levels were decreased to or even less than control values in all cases (Fig. 5A). The positive effect of the drug on recessive OI cellular homeostasis was further confirmed by the reduction of ER cisternae size, as evaluated by transmission electron microscopy (Fig. 5B). No rescue of CRTAP and CyPB was instead detected after the treatment in the patients with normal transcript level, indicating that the 4-PBA effect is not due to rescue of folding of the mutant proteins (Fig. 5C).

### 4-PBA chaperone function rescues recessive OI cell homeostasis

**Fig. 6.**
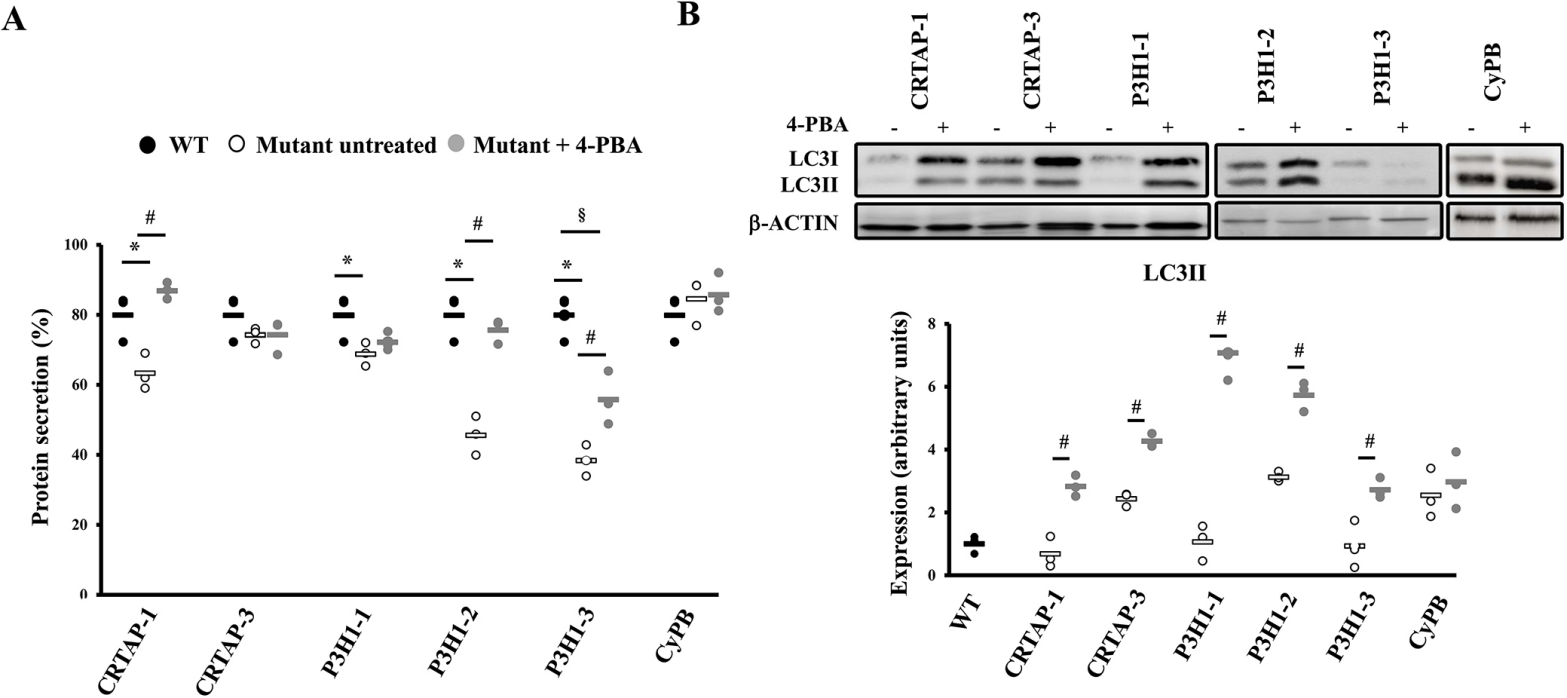
**4-PBA stimulates protein secretion and autophagy.** (A) Dot plot representing the amount of general protein secreted in the absence or presence of 4-PBA treatment in WT and OI patient fibroblasts. In the samples in which protein secretion was impaired in the basal condition, it was rescued by 4-PBA treatment. (B) Representative western blot to evaluate LC3 expression (top) in control (WT) and patient cells in the absence (−) and presence (+) of 4-PBA, and dot plot of the quantitative analysis (bottom). An increase of LC3-II levels in cells after 4-PBA treatment was detected. *P<0.05 mutant fibroblasts with respect to control fibroblasts. ^#^*P*<0.05 treated mutant fibroblasts with respect to untreated mutant fibroblasts. ^§^*P*<0.05 treated mutant fibroblasts with respect to untreated control fibroblasts. β-actin was used for normalization. WT untreated values are shown as black dots, mutants untreated as white dots and mutants treated as gray dots.

In order to determine the mechanism of action of 4-PBA, we investigated in our system the effect of the drug on collagen secretion and on general protein secretion. Collagen secretion was unaffected by the treatment, as were collagen post-translational modifications (data not shown). However, protein labeling with ^35^S-L-methionine and ^35^S-L-cysteine revealed an increased total protein secretion upon 4-PBA administration in all the cells tested in which it was severely affected in the basal condition, namely CRTAP-1, P3H1-1, P3H1-2 and P3H1-3 (Fig. 6A), indicating its chaperone activity.

**Fig. 7.**
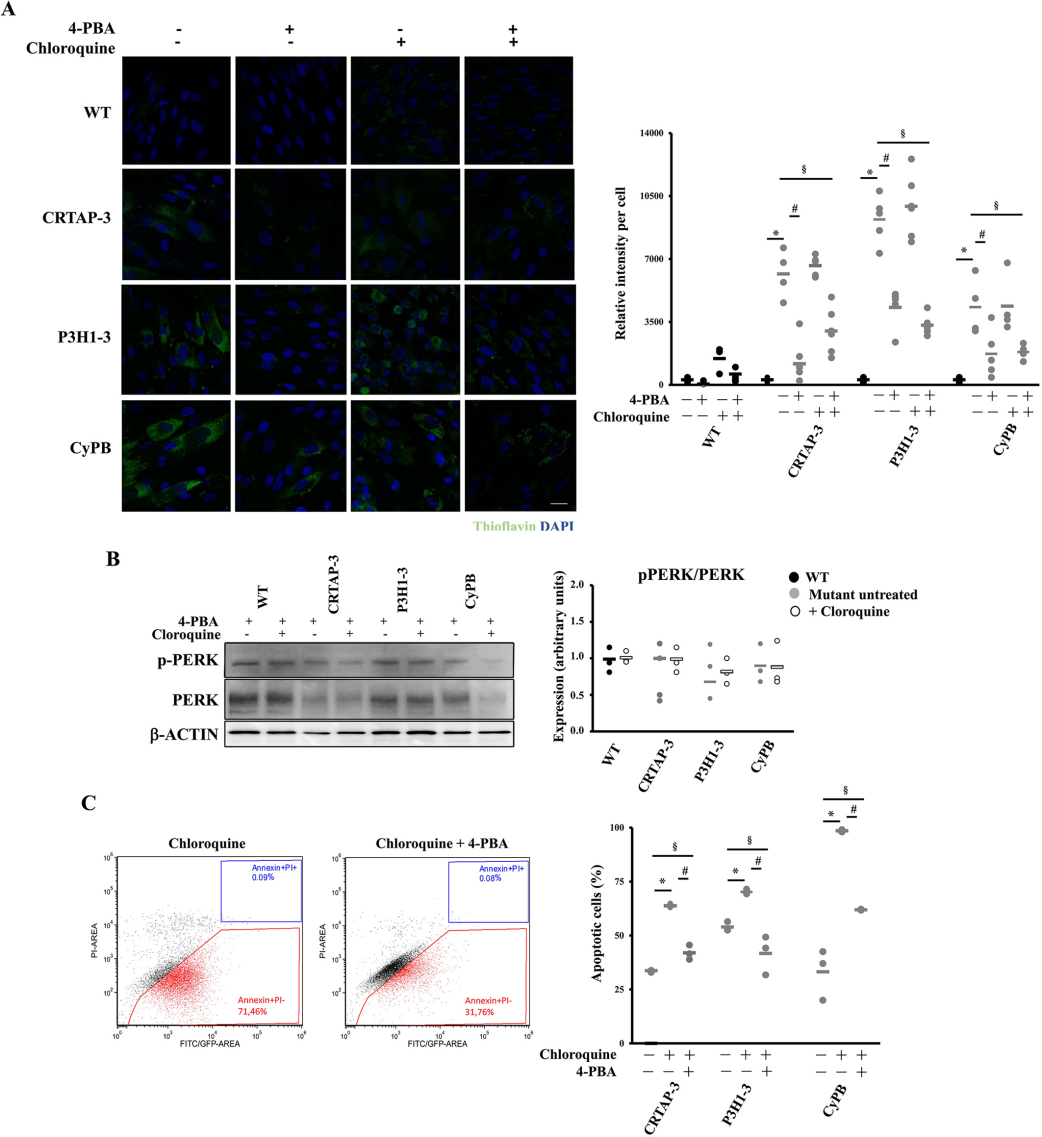
**4-PBA chaperone function is mainly responsible for homeostasis rescue in the recessive OI cells.** (A) ER proteostasis was evaluated using thioflavin T (ThT). Representative immunofluorescence images are shown on the left and the fluorescence quantitation graph is reported on the right. Mutant cells revealed an increased fluorescence compared to control, indicating the intracellular accumulation of misfolded proteins. 4-PBA treatment significantly reduced the ThT fluorescence, proving its chaperone role also following autophagy inhibition with chloroquine. WT is shown in black, mutants in gray. (B) Representative western blot to evaluate pPERK:PERK expression in the absence (−) and presence (+) of 4-PBA and of chloroquine and quantitative analysis. 4-PBA normalized the p-PERK:PERK ratio even when autophagy was impaired. β-actin was used for normalization. WT untreated values are shown as black dots, mutants untreated as gray dots, cells treated with chloroquine as white dots. (C) Representative FACS plots and quantitative analysis of the fraction of apoptotic events following staining with annexin V (FITC) and propidium iodide (PI) in the absence (−) and presence (+) of chloroquine and of 4-PBA. Even when autophagy was impaired, 4-PBA decreased apoptosis. *P<0.05 mutant fibroblasts treated with chloroquine with respect to untreated. ^#^*P*<0.05 mutant fibroblasts treated with 4-PBA and chloroquine with respect to treated with chloroquine. ^§^*P*<0.05 mutant fibroblasts treated with 4-PBA and chloroquine with respect to untreated fibroblasts. Scale bar: 20 µm.

Interestingly, an increased LC3-II level in all mutant cells treated with 4-PBA was detected, clearly supporting a 4-PBA stimulatory effect on autophagy in OI recessive cells (Fig. 6B). In order to determine whether the rescue of the cellular homeostasis following 4-PBA treatment was due to its chaperone function or to its autophagy-stimulating ability, ER proteostasis, PERK activation and cell survival were monitored in the absence or presence of chloroquine, a pharmacological inhibitor of autophagy. Thioflavin T (ThT), a small molecule that exhibits increased fluorescence when it binds to protein aggregates, was used to quantify ER proteostasis (Beriault and Werstuck, 2013). Enhanced ThT fluorescence was detectable in mutant cells compared to control, indicating the accumulation of intracellular misfolded material (Fig. 7A). 4-PBA treatment significantly reduced the ThT fluorescence, proving the reduction of protein accumulation (Fig. 7A). Importantly, this effect of 4-PBA was evident also when inhibiting autophagy with chloroquine (Fig. 7A). Furthermore, the p-PERK:PERK ratio and apoptosis were decreased by 4-PBA when autophagy was impaired, finally corroborating the primary chaperone function of the drug in rescuing cell homeostasis (Fig. 7B,C).

To evaluate whether other chemical chaperones could have a similar effect on OI cells, tauroursodeoxycholic acid (TUDCA), approved for cholestasis (Wagner and Trauner, 2016), was used. TUDCA did not show any effect on p-PERK, apoptosis and autophagy levels, thus suggesting a specificity of 4-PBA in the rescue of recessive OI fibroblasts homeostasis (Fig. S3).

## DISCUSSION

In the past 10 years, the prolyl-3-hydroxylation complex has been demonstrated to be crucial for proper type I collagen folding and post-translational modifications (Marini and Blissett, 2013). Mutations in any of its components, CRTAP, P3H1 and CyPB, are associated with recessive forms of moderate to lethal OI, characterized by the presence of abnormal ECM and impaired mineralization associated with bone fragility (Marini et al., 2017). Here, we move our attention from the extra- to the intracellular space and describe the effect of overmodified type I collagen on cellular homeostasis of seven recessive OI cases, three carrying mutations in *CRTAP*, three in *P3H1* and one in *PPIB*, using skin fibroblasts in which no mutant protein was detectable (Fig. 1C).

### Overmodified type I collagen in recessive OI mutants causes UPR and apoptosis activation

In all cell lines, the presence of overmodified collagen was demonstrated by metabolic labeling and electrophoretic analysis (Fig. 2A). Likely due to the increased accumulation of type I collagen molecules in the ER, cisternae enlargement and cellular vacuolization were detected (Fig. 5B). Interestingly, in the presence of similar broadening of the α-bands, significant intracellular collagen retention and ER enlargement was not clearly evident in P3H1-2 cells, suggesting a variable ability of the cells to handle overmodified type I collagen, either due to a different level of collagen overmodification or to the effect of modifiers affecting collagen secretion (Fig. 2, Fig. 5B). Indeed, although collagen electrophoretic analysis is a quick and simple tool to reveal post-translational overmodifications, it does not allow the detection of subtle differences (Barnes et al., 2006; Cabral et al., 2007) that have been previously demonstrated in OI patients and may potentially impact on protein secretion (Amor et al., 2011; Barnes et al., 2006; Marini et al., 2007; Taga et al., 2013). Furthermore, the functional role of collagen O-glycosylation is not clearly defined yet and, if some information is available regarding its possible extracellular effect on increasing collagen stability against proteolytic degradation, control of lateral growth of the fibrils, interaction with non-collagenous proteins and the cross-linking process, nothing has been reported so far on its intracellular effect (Perdivara et al., 2013).

Following collagen folding in the ER, its secretion requires the assembly of specific large COPII vesicles, whose formation depends on a large number of proteins and lipids. Thus, it is not surprising that the complex collagen secretory machinery may be tuned by the action of modifiers (Vollmann et al., 2017).

To maintain the functional integrity of ER under stress conditions, the evolutionarily conserved adaptive response, the UPR, is generally turned on. Indeed, UPR activation affecting cell homeostasis and likely modulating disease severity was reported in the presence of mutations in ECM molecules, including the fibrillar collagen type II and X (Boot-Handford and Briggs, 2010). In recessive OI cells, the PERK branch of the UPR is activated, as demonstrated by an increased p-PERK:PERK ratio and upregulation of its effector ATF4 (Fig. 3C). In the presence of ER stress, BIP, the master regulator of activation of the UPR branches, is released from the UPR sensors to favor protein folding and this event activates the specific ER cellular response. Interestingly, three out of seven OI cell lines did not show upregulation of BIP, hinting at other regulatory proteins being involved in the ER stress response in the presence of overmodified type I collagen retention, as suggested in previous reports (Besio et al., 2018; Forlino et al., 2007; Mirigian et al., 2016). UPR activation is not sufficient for cell homeostasis and CRTAP-2, CRTAP-3, P3H1-2 and CyPB recessive OI fibroblasts also showed upregulated autophagy (Fig. 4), which is often activated to regulate the lysosome-dependent turnover of cell materials to reduce the ER overload (Galluzzi et al., 2017; Green and Levine, 2014). Surprisingly, autophagy was also stimulated in P3H1-2 cells in which no reduction of collagen secretion, no UPR activation and no ER cisternae enlargement were detectable. The splice-site mutation in this cell line (c.1223+2T>G), predicted to cause exon 7 removal, should result in the translation of a shorter P3H1 (p.Asp391Valfs46) that could indeed be eliminated through autophagy. Nevertheless, the strong reduction of RNA expression demonstrated the activation of nonsense-mediated decay, likely minimizing the amount of protein synthesis. Despite autophagy activation, apoptosis was promoted in all analyzed cell lines (Fig. 3A).

### Common pathways are activated in recessive and classical forms of OI

The reported overmodified collagen retention, UPR activation and cell death found in recessive OI type VII, VIII and IX resemble what was previously detected in the OI forms due to mutations in *COL1A1* and *COL1A2* genes and characterized by the synthesis of structurally altered collagen molecules. In particular, in fibroblasts from patients with classical OI forms, we recently demonstrated that the intracellular-retained overglycosylated collagen causes ER cisternae enlargement, and the inefficiency of the UPR to counteract the constitutive synthesis of mutant collagen brought cells to death (Besio et al., 2018). In our system, we also described autophagy activation as a general mechanism associated with *COL1A1* mutations, but detectable only in few cases with mutations in *COL1A2* (Besio et al., 2018). We hypothesized that endogenous autophagy in OI cells could be linked to the presence of a higher amount of mutant collagen since, due to its stoichiometry, 75% of trimers will be overmodified in the presence of mutant α1, versus 50% when α2 chains are mutated. Interestingly, in the analyzed recessive cells, in which all the collagen type I is overmodified, not all patients had the same autophagic response. Although we cannot exclude a mutation or site-dependent effect of structural defects in collagen chains, these new results support the hypothesis of a role for modifiers in modulating cell response to stress. Indeed, in a previous study using skin and bone samples from the Brtl mouse, a model for dominant OI carrying a G349C substitution in α1(I) and either a moderate or a lethal outcome, we found in mice with non-lethal outcome a better ability to react to mutant collagen retention. Such capacity was associated with an increased expression of chaperone proteins (Bianchi et al., 2012; Forlino et al., 2007). Some years later, we confirmed a different ability to manage cell stress and thus to guarantee cell homeostasis in patients cells carrying identical mutation, but different outcome (Besio et al., 2018).

Of note, independently from the autophagic cellular response, apoptosis is upregulated in both dominant and recessive mutant cells. Thus, apoptotic pathway activation represents a hallmark of unsolved cell stress, both in dominant and in recessive forms of the disease (Besio et al., 2018; Bianchi et al., 2012; Galluzzi et al., 2017; Green and Levine, 2014; Mirigian et al., 2016). P3H1-2 represents an exception to this rule since, in this cell line, apoptosis is not activated by sustained ER stress due to mutant collagen retention, but possibly as a consequence of sustained autophagy.

### 4-PBA: a potential common therapy for recessive and classical forms of OI

The identification of novel targets for disease treatment is of valuable significance to develop novel therapies. The recognition of altered pathways common to several diseases is even more relevant for rare diseases with a limited number of affected patients. We previously proved that 4-PBA successfully ameliorates classical OI cell homeostasis *in vitro* using OI patients' fibroblasts, and bone phenotype *in vivo* using the OI zebrafish model *Chihuahua* (Besio et al., 2018; Gioia et al., 2017). The drug activated autophagy and increased general protein secretion in OI dominant fibroblasts, and improved bone mineralization and bone histomorphometric parameters in the zebrafish model. 4-PBA is an FDA-approved drug for urea cycle disorders; thus, its repositioning for a different disease will definitely speed up the bench-to-bedside transition (Matoori and Leroux, 2015). Nevertheless, the multiple recognized functions of 4-PBA need to be taken into consideration for a proper data interpretation. Associated to its ammonia scavenger role, 4-PBA has a recognized chaperone function, favoring ER protein folding and thus attenuating the UPR in the presence of ER stress caused by misfolded protein accumulation (Pettit and Fellner, 2014), and it acts as a histone deacetylase inhibitor, modulating chromatin accessibility and thus gene expression (Butchbach et al., 2016). Interestingly, at least in yeast cells, 4-PBA attenuates the UPR by accelerating the degradation of the ER-stress sensor Ire1, rather than by restoring the global protein folding; indeed, UPR attenuation was detectable even in the absence of ER stress (Daosukho et al., 2007; Mai et al., 2018).

Here, we dissected whether the positive effect of 4-PBA was due to its autophagy-stimulating ability or to its chaperone function, and finally proved its relevance in assisting protein secretion.

Taking all these findings into consideration, our results prove and extend the potential use of 4-PBA as chemical chaperone to the OI forms characterized by overmodified collagen production. For the first time, we demonstrated the potential pharmacological benefit of this drug for the recessive forms of OI with defects in the 3-hydroxylation complex. In almost all the analyzed fibroblasts carrying mutations in the P3H1 complex, 4-PBA administration reduced PERK activation and decreased apoptosis (Fig. 5A). The improved general protein secretion detected in the recessive OI resembles the findings described in dominant cases. The restoration of normal ER cisternae size (Fig. 5B) and the reduced ThT fluorescence (Fig. 7A) further supports the reduction of misfolded protein accumulation.

Of note, treatment of CyPB mutant cells seems to act in a different way. Drug administration reduced apoptotic death, but neither autophagy nor protein secretion were significantly augmented. CyPB is involved in other intracellular complexes. It is a binding partner for lysyl hydroxylase isoforms, thus affecting collagen hydroxylation and crosslinks, and interacts with BIP and PDI, thus having a relevant role in the folding of the collagen C-propeptides and on the kinetics of collagen chain association (Gruenwald et al., 2014; Heard et al., 2016; Terajima et al., 2016). Indeed, *PPIB-*null cells showed a delay in trimer association together with the increased post-translational modification present also in *CRTAP*- and *P3H1*-null cells (Pyott et al., 2011; van Dijk et al., 2009). With the limit of the use of a single cell line, mainly due to the extreme rarity of OI type IX, we can hypothesize that the multiple roles of CyPB may be differentially and specifically affected/modulated by 4-PBA treatment, and further experiments will be necessary to shed light on the mechanism.

### Study limitations

As a cellular model, we used primary fibroblasts from recessive OI patients. Since skin biopsy has limited invasiveness, a large body of literature is available on OI biochemical characterization based on this cell type, and fibroblasts share with osteoblasts the production of a high amount of collagen type I and several biochemical pathways (Bianchi et al., 2012). Furthermore, a skin phenotype is often described in OI patients (Balasubramanian et al., 2016). Nevertheless, OI is mainly a bone disorder and the bone-forming cells are known to produce even higher amounts of collagen type I, with a higher glycosylation level compared to fibroblasts (Sarafova et al., 1998). Indeed, how osteoblasts react to mutant collagen retention has been recently addressed using calvarial osteoblasts from the OI knock-in murine model α2(I)-G610C. The misfolded procollagen was found accumulated in the ER, causing an unusual cell stress, which was neither activating a conventional UPR nor causing ER overload, although EIF2α was found phosphorylated (Mirigian et al., 2016). Interestingly, in a more recent paper using the same OI murine model, ER-stress-related genes were found upregulated in hypertrophic chondrocytes expressing type I collagen (Scheiber et al., 2019). Further investigation in different murine models and, likely, in human osteoblasts are necessary.

For proper interpretation of our results, it should also be considered that the *in vitro* growth and expansion of the cells could have imposed an artificial ‘stress’ that we cannot exclude to have some effects on the activation of specific UPR branches. Anyway, all controls and mutant cell lines were similarly expanded, likely supporting the truthfulness of the described differences. To properly translate *in vitro* data to patients, *in vivo* validation is needed. We recently demonstrated in osteoblasts from the OI zebrafish model *Chihuahua* an ER cisternae enlargement associated with mutant collagen type I synthesis and we proved that 4-PBA was indeed restoring ER cisternae size, likely favoring collagen secretion (Gioia et al., 2017). The identification of the involved pathways in mammals needs further investigation.

In conclusion, we identified ER stress as a common potential target for the treatment of recessive OI carrying mutations in components necessary for collagen post-translational modifications and for the cure of classical dominant OI. The finding that the same chemical chaperone is effective in cells synthesizing overmodified collagen increases the potential clinical use of 4-PBA for multiple OI forms.

## MATERIALS AND METHODS

### Human fibroblasts

Seven human primary dermal fibroblasts from skin biopsies of OI patients carrying mutations in one of the genes coding for the three members of the collagen prolyl 3-hydroxylation complex – *CRTAP* (CRTAP-1, CRTAP-2, CRTAP-3) (Amor et al., 2011; Caparros-Martin et al., 2013; Valli et al., 2012), *P3H1* (P3H1-1, P3H1-2 and P3H1-3) (Caparros-Martin et al., 2013) and *PPIB* (CyPB) (Caparros-Martin et al., 2013) – and three pediatric controls (Promo Cell) were obtained after informed consent and used up to passage 10 (P10) (Table 1). Cells were grown at 37°C in humidified atmosphere containing 5% CO_2_ and cultured in Dulbecco's modified Eagle's medium (DMEM; 4.5 g/l glucose; Lonza) supplemented with 10% fetal bovine serum (FBS; Euroclone), 4 mM glutamine (Euroclone), 100 µg/ml penicillin and streptomycin (Euroclone). No ascorbic acid was added to expansion media. For each experiment, except where differently stated, 2.5×10^4^ cells/cm² were plated and harvested after 5 days with no media change. For drug treatment, cells were incubated for 15 h with 5 mM 4-PBA (Sigma-Aldrich) or with 0.96 mM TUDCA (Sigma-Aldrich). The lysosome fusion with autophagosome was blocked using 10 µM chloroquine (Sigma-Aldrich) for 6 h.

### Sequencing

Genomic DNA from P3H1-1 was extracted from fibroblasts by standard procedure. Exons were amplified by PCR and Sangers' sequencing was performed.

### Expression analysis

Total RNA was extracted from patients’ fibroblasts using TriReagent (Sigma-Aldrich) according to the manufacturer's protocol. DNase digestion was performed using the Turbo DNA Free Kit (Ambion, Applied Biosystems), and RNA integrity was verified on agarose gel. cDNA was synthetized and qPCR was performed on the Mx3000P Stratagene thermocycler using Syber Green Master Mix (Applied Biosystems) with custom primers. For *CRTAP* (NM_006371.4) the forward primer was 5′-CCCAGACCTGAAGCAGTT-3 (nt 1180-1197) and the reverse primer was 5′-TTCTCCCTCATCATCATCCATT-3′ (nt 1278-1257). The *PPIB* (NM_000942 .4) forward primer was 5′-GGAGAGAAAGGATTTGGCTAC-3′ (nt 413-433) and the reverse primer was 5′-CAGGCTGTCTTGACTGTCGTGA-3′ (nt 651-630). The *P3H1* (NM_001243246.1) forward primer was 5′-CGGGTGGCTGGCGGTTCCG-3′ (nt 78-96) and the reverse primer was 5′-ACCTCGGCTTGGGAGGCAGC-3′ (nt 184-165). All reactions were performed in triplicate. *GAPDH* was used as normalizer. The *GAPDH* (NM_002046.5) forward primer was 5′-ATACCAGGAAATGAGCTTGACAAA-3′ (nt 1035-1057) and the *GAPDH* reverse primer was 5′-TCCTCTGACTTCAACAGCGACAC-3′ (nt 1130-1107). Relative expression levels were calculated using the ΔΔCt method.

### Protein lysates

Fibroblasts were washed and scraped in PBS, centrifuged at 1000 ***g*** for 4 min, lysed and sonicated in RIPA buffer (150 mM NaCl, 1% IGEPAL^®^ CA-630, 0.5% sodium deoxycholate, 0.1% SDS, and 50 mM Tris, pH 8) supplemented with protease inhibitors (13 mM benzamidine, 2 mM N-ethylmalemide, 5 mM ethylenediaminetetraacetic acid, 1 mM phenylmethylsulfonyl fluoride and 2 mM NaVO_3_). Proteins were quantified by RC DC Protein Assay (Bio-Rad). Bovine serum albumin (BSA) (Sigma-Aldrich) was used as standard.

### Western blot

Proteins from human fibroblast lysates (10-50 µg) were separated on SDS-PAGE with acrylamide percentage ranging from 6 to 15%, depending on the size of the analyzed protein (Table S1). The proteins were electrotransferred to a PVDF membrane (GE Healthcare) at 100 V for 2 h on ice in 19 mM Tris-HCl, 192 mM glycine and 20% (v/v) methanol. The membranes were then blocked with 5% (w/v) BSA in 20 mM Tris-HCl, 500 mM NaCl, pH 7.5 (TBS), 0.05% (v/v) Tween-20 (Sigma-Aldrich) (TBS-T) at room temperature (RT) for 1 h. After washing with TBS-T, the membranes were incubated with 1:1000 primary antibody against the specific proteins CRTAP (generously provided by Dr Lee Brendan, Baylor College of Medicine,TX, USA), P3H1 (NovusBio), CyPB (Proteintech), BIP (Cell Signaling), PERK (Cell Signaling), PDI (Cell Signaling), p-PERK (Thr980; Cell Signaling), LC3A/B (Cell Signaling), cleaved caspase-3 (Cell Signaling), ATF4 (Novus Biological), ATF6 (Abcam) in 5% BSA in TBS-T overnight at 4°C. The appropriate secondary antibody anti-mouse (Cell Signaling), anti-rabbit (Cell Signaling) or anti-goat (Santa Cruz Biotechnology) was added at dilution of 1:2000 in 5% BSA in TBS-T for 1 h at RT. Anti-β-actin antibody (Santa Cruz Biotechnology) diluted 1:1000 in 5% BSA in TBS-T was used for protein loading normalization. The signal was detected by ECL western blotting detection reagents (GE Healthcare) and images were acquired with ImageQuant LAS 4000 (GE Healthcare), using the ImageQuant LAS 4000 1.2 software. Band intensities were evaluated by densitometry, using ImageQuant TL analysis software. For each gel, the intensity of the control band was set equal to one, and the expression of the mutant samples was expressed as fold difference. For each cell line, three independent lysates were collected and technical triplicates were performed.

### Collagen analysis

Labeling of collagen with L-[2,3,4,5-^3^H]-proline (PerkinElmer) was used to evaluate collagen overmodification and secretion. A total of 2.5×10^4^ fibroblasts/cm^2^ were plated into 6-well plates and grown for 24 h. Cells were then incubated for 2 h with serum-free DMEM containing 4 mM glutamine, 100 µg/ml penicillin and streptomycin, and 100 µg/ml (+)-sodium L-ascorbate (Sigma-Aldrich) to stimulate collagen production. For steady-state experiments, the labeling was performed for 18 h in the same media using 28.57 µCi of ^3^H-Pro/ml. For chase experiments, the labeling was performed for 4 h using 47.14 µCi of ^3^H-Pro/ml, then the labeling media was replaced with serum-free DMEM containing 2 mM proline (Sigma-Aldrich), 4 mM glutamine, 100 µg/ml penicillin and streptomycin, and 100 µg/ml (+)-sodium L-ascorbate (chase media). Collagen was collected at 0.5, 1, 2 and 3 h after the chase. Collagen extraction was performed as previously reported (Forlino et al., 2019; Valli et al., 1991). Briefly, medium and cell lysate fractions were digested overnight with 100 ng/ml of pepsin in 0.5 M acetic acid at 4°C. Collagen was then precipitated using 2 M NaCl, 0.5 M acetic acid. Collagen was resuspended in Laemmli buffer (62 mM Tris-HCl, pH 6.8, 10% glycerol, 2% sodium dodecyl sulfate, 0.02% Bromophenol Blue) and the radioactivity [counts per minute (CPM)] was measured using a liquid scintillation analyzer (PerkinElmer TRI-CARB 2300 TR).

For steady-state analyses, equal amounts of ^3^H-labeled collagen from each patient’s cells were loaded on 6% urea-SDS gels in a non-reducing condition. For chase analyses, the same volume of ^3^H-labeled collagens from each time point was electrophoresed. The gels were fixed in 45% methanol, 9% glacial acetic acid, incubated for 1 h with enhancer (PerkinElmer, 6NE9701), washed in deionized water and dried. ^3^H gel radiographs were obtained by direct exposure of dried gels to hyperfilm (Amersham) at −80°C. The radiography films were digitalized by VersaDoc 3000 (Bio-Rad). To quantify the intracellular collagen retention, the ratio between the CPM in the cell layer and the CPM in medium plus cell layer was evaluated. To quantify the percentage of collagen secretion, the ratio between the density of the α1(I) band in the media and the total collagen (medium plus cell layer) was evaluated by Quantity One software (Bio-Rad) (Ciccocioppo et al., 2013).

To analyze the effect of 4-PBA on collagen secretion, cells were labeled for 18 h in the absence or presence of 5 mM 4-PBA using 28.57 µCi of ^3^H-Pro/ml. Collagen extraction from the media was performed as previously reported and an equal volume was loaded on the SDS-urea-PAGE. The ratio between the density of the α1(I) band in the medium was evaluated on the digitalized fluorography, and the value was normalized to the DNA extracted from the cell layer.

### Transmission electron microscopy analysis

For transmission electron microscopy analysis, fibroblasts from controls and patients were trypsinized and centrifuged at 1000 ***g*** for 3 min. The pellet was fixed with 1% glutaraldehyde in the culture medium for 2 h at RT. The cells were rinsed in PBS and then in H_2_O. Finally, the fibroblasts were fixed in 2% (w/v) OsO_4_ in H_2_O for 2 h at RT, rinsed in distilled water and embedded in 2% agarose. The specimens were dehydrated in acetone and finally infiltrated with epoxy resin overnight and polymerized in gelatin capsules at 60°C for 24 h. Thin sections (60-70 nm thick) were cut on a Reichert OM-U3 ultramicrotome with a diamond knife and collected on 300-mesh nickel grids. The grids were stained with saturated aqueous uranyl acetate by lead citrate and observed with a Zeiss EM900 electron microscope, operated at 80 kV with objective aperture of 30 µm.

### XBP1 splicing analyses

cDNA from control and patients cells was used for PCR amplification across the region of the *XBP1* cDNA (NM_005080.3) containing the intronic target of IRE1α ribonuclease using 0.3 μM sense (nt 396-425; 5′-TCAGCTTTTACGAGAGAAAACTCATGGCCT-3′) and antisense (nt 696-667; 5′-AGAACATGACTGGGTCCAAGTTGTCCAGAA-3′) primers. Following a 30 min incubation at 50°C, reactions were cycled 30 times at 94°C, 60°C and 72°C for 30 s at each temperature. Reaction products were electrophoresed on 8% TBE acrylamide gels and visualized by ethidium bromide staining.

### LC3 immunofluorescence

A total of 1.5×10^4^ fibroblasts were plated on sterile glass coverslips (Marienfeld) in 24-well plates in triplicate. After 5 days, cells were treated for 6 h with 10 μM chloroquine. Following the treatment, the medium was removed and cells were fixed with cold 100% CH₃OH for 15 min at −20°C, washed three times with PBS and blocked for 1 h in 1% BSA in PBS containing 0.3% Triton X-100. Then, cells were incubated with LC3 primary antibody (Cell Signaling) diluted 1:500 in 1% BSA, 0.3% Triton X-100 in PBS overnight at 4°C. Cells were washed three times with PBS and incubated with secondary antibody [Alexa-Fluor-488-conjugated F(ab′) fragment anti-rabbit IgG, Immunological Sciences] diluted 1:2000 in 1% BSA, 0.3% Triton X-100 in PBS for 2 h at RT. Nuclei were stained with 4′,6-diamidino-2-phenylindole (DAPI; Sigma-Aldrich). The samples were analyzed using an SP5-Leica confocal microscope (Leica). The total area of punctate signal per cell was measured by the Leica software LAS4.5.

### Thioflavin-T labeling

A total of 1.5×10^4^ fibroblasts were plated on sterile glass coverslips (Marienfeld) in 24-well plates. After 4 days, cells were incubated with 5 μM Thioflavin T (ThT; Sigma-Aldrich) for 15 h in the presence or absence of 4-PBA and in the presence or absence of chloroquine. The medium was removed and cells were fixed with 4% paraformaldehyde in PBS for 20 min at RT. Nuclei were stained with DAPI (Sigma-Aldrich). The samples were analyzed using an SP8-Leica confocal microscope (Leica). The excitation and emission settings were: DAPI (Ex. MP laser 800 nm, Em. 410-530 nm), ThT (Ex. 458 nm, Em. 480-520 nm). The total area of punctate signal per cell was measured using the Leica software LAS4.5.

### Fluorescence activated cell sorting (FACS)

To analyze apoptosis, the FACS Annexin V/Dead Cell Apoptosis Kit (Invitrogen) was used following the manufacturer’s instructions. As positive control for the activation of apoptosis, cells were treated with 20 μM thapsigargin (Sigma-Aldrich) for 24 h in serum-free DMEM. Samples were analyzed by Cell Sorter S3 (Bio-Rad); 1×10^4^ events for each sample were considered measuring the fluorescence emission at 510-540 nm and >565 nm. For autophagy inhibition, 50 µM chloroquine was used.

### Protein secretion

OI patients' fibroblasts were plated in 24-well plates and labeled with 5 µCi/ml [^35^S] EXPRESS35S Protein Labeling Mix (PerkinElmer) in DMEM without L-methionine, L-cystine and L-glutamine for 1 h at 37°C_._ Total proteins from medium and cell layer were precipitated with 10% trichloroacetic acid. Proteins were washed with acetone twice and resuspended in 60 mM Tris-HCl, pH 6.8, 10% sodium dodecyl sulphate. The radioactivity (CPM) of the samples was measured using a liquid scintillation analyzer (TRI-CARB 2300 TR). The percentage of protein secretion was calculated based on the ratio between the CPM in the media and the CPM in medium and cell layer, evaluated in five technical replicates.

### Statistical analysis

Statistical differences between patients and controls were evaluated by two-tailed Student's *t*-test. Statistical differences between controls, patients and treated patients and between the different treatments were evaluated by one-way ANOVA using Sigma plot 11.0 (Fisher). All data passed tests for normality and equal variance. Technical triplicates were performed and values were expressed as mean±s.d. A *P*-value <0.05 was considered significant.

## Supplementary Material

Supplementary information
