# Supplementary material for: Cellular stress due to impairment of collagen prolyl hydroxylation complex is rescued by the chaperone 4-phenylbutyrate
Source: Dis Model Mech. 2019 Jun 20;12(6):dmm038521. doi: 10.1242/dmm.038521 (PMC6602311; doi:10.1242/dmm.038521)
Supplement: Supplementary information [file dmm-12-038521-s1.pdf]

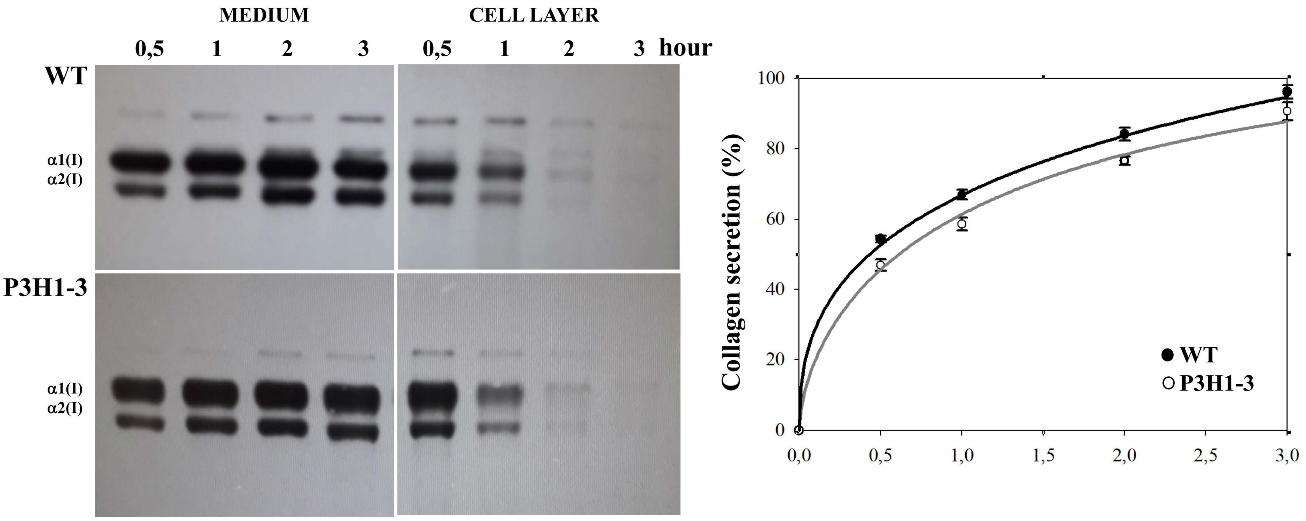

Fig. S1. Collagen secretion in mutant cells. Type I collagen secretion was evaluated by incubating the cells with ^{3}H-Pro and extracting the collagen from both medium and cell layer fractions at the indicated time points. Samples were run on SDS-PAGE and the ratio between the densitometric value of collagen α1(I) in the media and the total collagen α1(I) (collagen present in medium and in cell layer) was evaluated and shown in the plot. Collagen secretion is delayed in the mutant cells tested compared to controls.

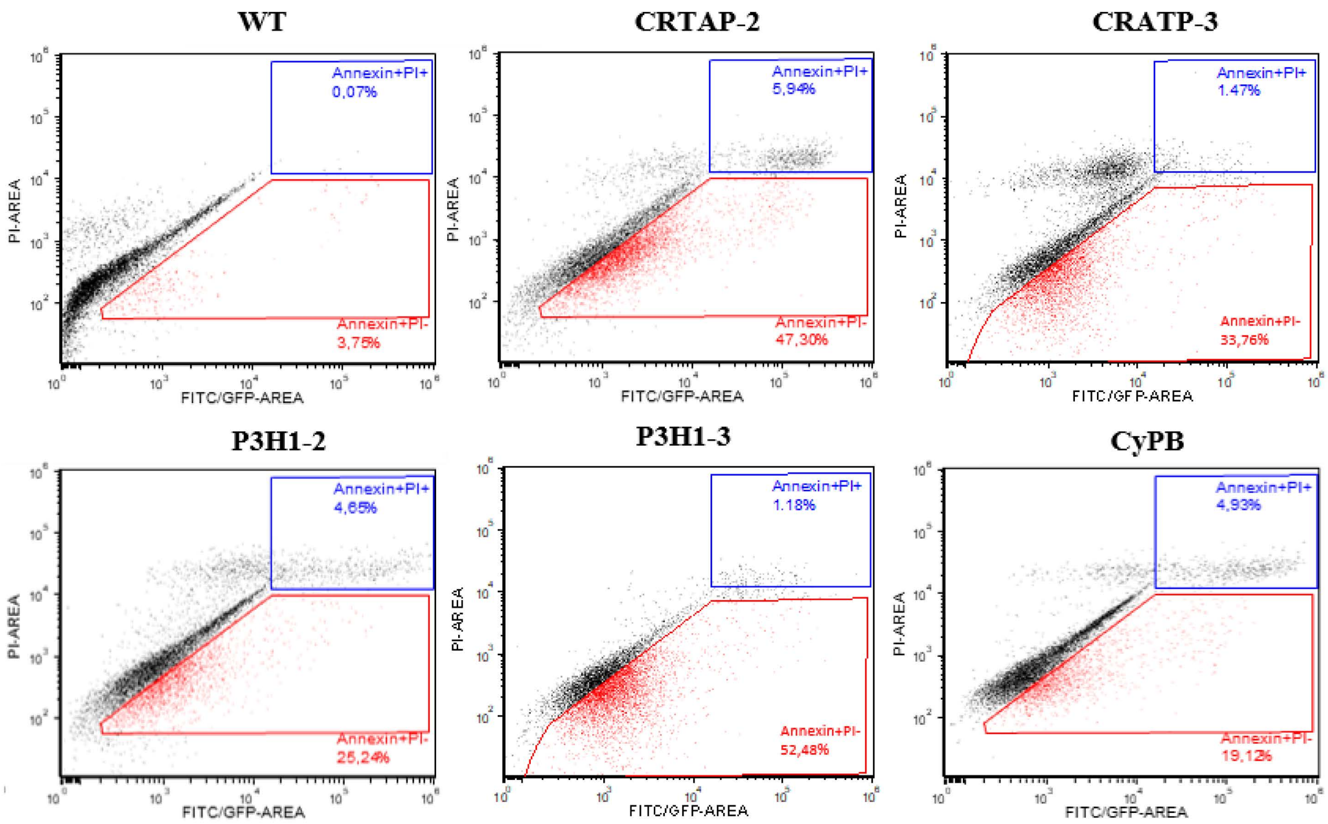

Fig. S2. FACS analysis of mutant cells. Representative plots of the fraction of apoptotic events in WT and OI patients' fibroblasts upon cells staining with annexin V (FITC) and propidium iodide (PI).

Disease Models & Mechanisms • Supplementary information

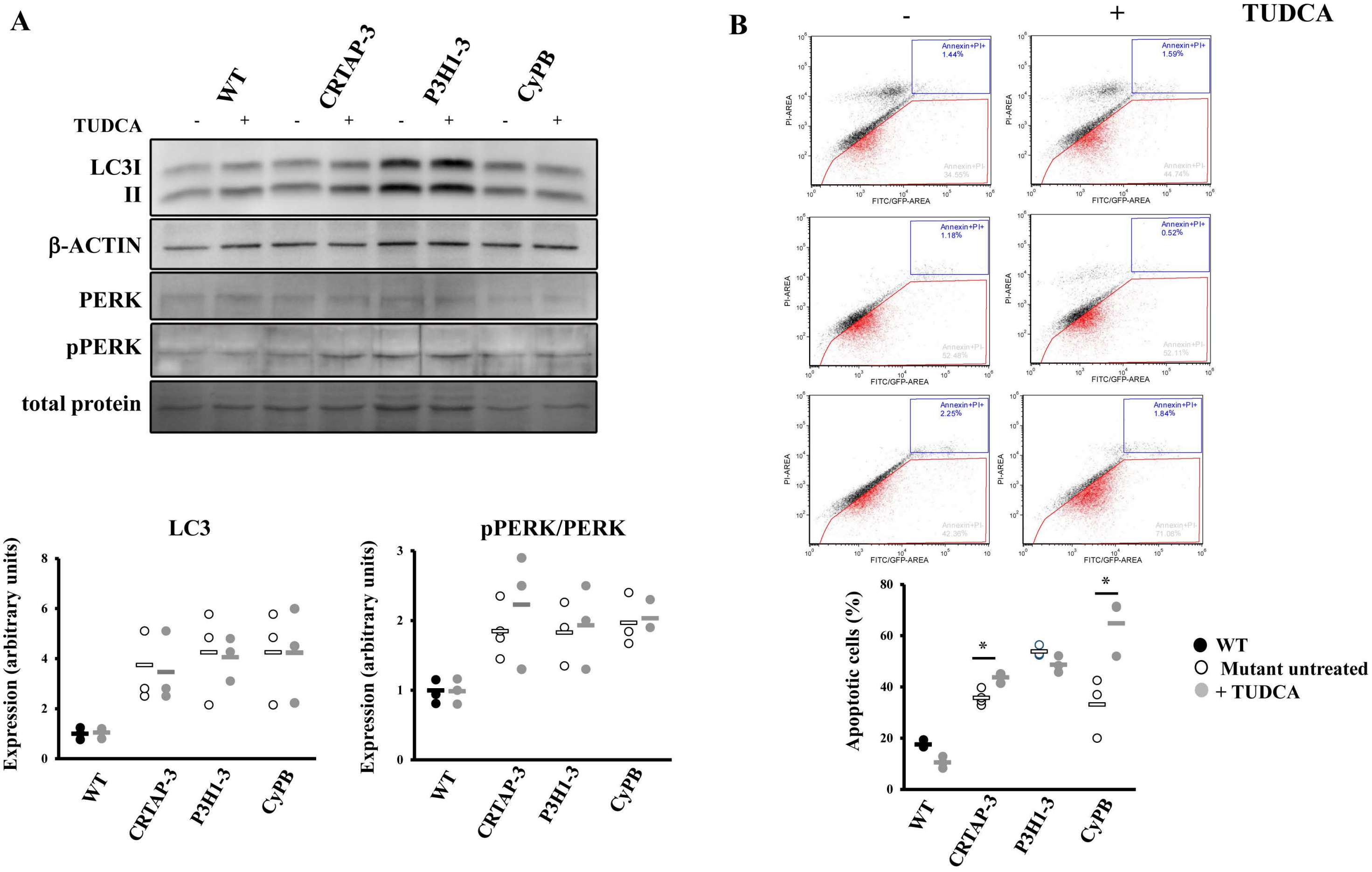

Fig. S3. TUDCA does not ameliorate recessive OI fibroblasts homeostasis. (**A**) Representative western blots (top) and quantitative analysis (bottom) of p-PERK and LC3II in absence (-) or in presence (+) of TUDCA in control (WT) cells and in cells with mutations in CRTAP, P3H1 and CyPB. The levels of these proteins were compared in treated versus untreated cells. p-PERK/PERK and LC3II levels were unchanged. β-actin is used for normalization. (**B**) Quantitative analysis of the fraction of apoptotic events in the cell lines following FACS analysis upon cells staining with annexin V (FITC) and propidium iodide (PI). TUDCA did not reduce the percentage of apoptotic cells in mutant lines. * $p<0.05$.

Table S1

Antibody	Company	Catalog Number	% Acrylamide running gel
BiP	Cell signaling	31777	10
PDI	Cell signaling	3501	10
PERK	Cell signaling	5683	6
P PERK	Cell signaling	3179S	6
ATF6	Abcam	ab122897	10
ATF4	Novus biological	NB100-8525	10
LC3	Cell signaling kit	12741	15
Cleaved CASP3	Cell signaling kit	9915	15
CyPB	Proteintech	11607-1-AP	15
P3H1	Abnova	H00064175-b01p	10
CRTAP	Brendan Lee lab	Brendan Lee lab	10

Disease Models & Mechanisms • Supplementary information
